# Single‐center experience of the Crohn's disease exclusion diet in the United States: A retrospective study

**DOI:** 10.1002/ncp.70122

**Published:** 2026-04-15

**Authors:** Mayah Greenfield, Caroline Salozzo, Wenya Chen, Jennifer Strople, Jeffrey Brown, Joseph Runde

**Affiliations:** ^1^ Robert Louis Katz & Manne Research Institute Summer Scholars Program, Northwestern Medicine Chicago Illinois USA; ^2^ Department of Clinical Nutrition Ann & Robert H. Lurie Children's Hospital of Chicago Chicago Illinois USA; ^3^ Stanley Manne Children's Research Institute, Ann & Robert H. Lurie Children's Hospital of Chicago Chicago Illinois USA; ^4^ Division of Gastroenterology, Hepatology & Nutrition, Ann & Robert H. Lurie Children's Hospital of Chicago Chicago Illinois USA; ^5^ Department of Pediatrics Feinberg School of Medicine Chicago Illinois USA

**Keywords:** Crohn's disease, Crohn's disease exclusion diet, dietary therapy, pediatrics

## Abstract

**Background:**

The Crohn's Disease Exclusion Diet (CDED) offers a dietary means of inducing and maintaining remission for patients with Crohn's disease (CD). Descriptions of efficacy have emerged primarily from Israel, Europe, and Canada. Here, we offer real‐world experience, from a tertiary care center in the United States.

**Methods:**

We conducted a retrospective chart review of patients receiving education on the CDED and those adopting the diet. Demographics, medication exposure, disease‐related complications, and reported side effects were collected. Corticosteroid‐free clinical remission (SF‐CR) was measured at weeks 6, 12, and 24 via the pediatric Crohn's Disease Activity Index (PCDAI). Biomarkers were collected at baseline and while on the diet.

**Results:**

Only 8% (*n* = 32) of children with CD had met with a dietitian regarding the CDED. Of those, 75% (*n* = 24) adopted the diet. A statistically significant change in PCDAI from baseline to weeks 6, 12, and 24 (*χ* ^2^(3) = 9.61, *P* = 0.02) was observed. At 24 weeks, SF‐CR was achieved in 9/11 subjects who remained on the diet, with a significant decrease in fecal calprotectin (Wilcoxon signed‐rank test *p* < 0.01, 95% CI). In seven subjects with pre/post endoscopic assessment, endoscopic response was demonstrated in 71%. Side effects were mild and included increased abdominal pain, diarrhea, and weight loss (37.5%, *n* = 9).

**Conclusion:**

In this single‐center experience of children with mild to moderate CD who adopted the CDED, adherence was a challenge. However, for those who were able to sustain the diet, the CDED offered a safe and effective means of clinical remission.

## Graphical abstract

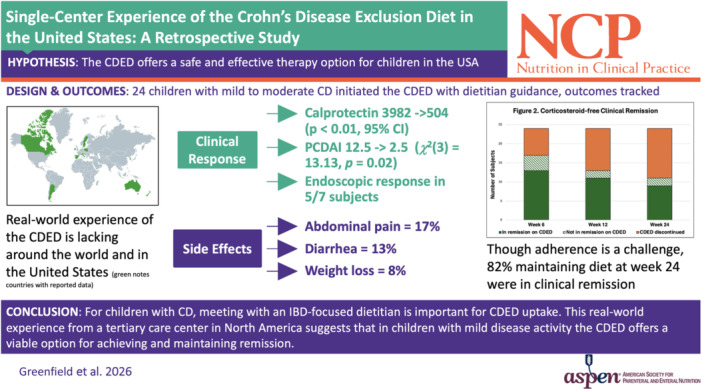

## INTRODUCTION

Despite advances in the management of Crohn's Disease (CD) with proactive monitoring strategies and increased options for medical therapy, treatment options remain limited. The risk of complications, including progression to surgery, remains high.^1^, ^2^ The incidence of CD continues to rise in countries across the globe^3^, ^4^ and data suggests that CD outcomes may be worse for those diagnosed as children.^4^, ^5^, ^6^ While novel mechanisms of action offer promise, medical therapy still relies on immune suppression and confers risk of sequelae with severe adverse effects and co‐morbidities.^7^, ^8^, ^9^ For this reason, diet‐based therapies that do not suppress immune function present a popular alternative for patients and their families.^10^, ^11^, ^12^

Exclusive enteral nutrition (EEN) has proven efficacy for induction of clinical remission and mucosal healing in pediatric subjects with CD.^13^, ^14^, ^15^, ^16^, ^17^ As such, EEN remains the gold standard for dietary therapy and should be considered the first line for induction of remission in patients with mild to moderate CD.^18^, ^19^

The Crohn's Disease Exclusion Diet (CDED), a 3‐phase whole‐foods based diet commonly initiated with partial enteral nutrition (PEN), is designed to offer the same benefit as EEN with the goal of improved adherence and the option to continue the diet as maintenance therapy.^20^, ^21^

Phase 1 (Weeks 0–6) includes 50% PEN with mandatory foods (chicken, eggs, bananas, apples, and potatoes) while limiting animal fats, soy, dairy, sugars, preservatives, foods rich in taurine (an amino sulfonic acid), and insoluble fiber. Of particular importance is decreasing exposure to emulsifiers, as they deplete the epithelial mucin layer, allowing bacterial penetration and triggering an immune response. Conversely, resistant starches (cooked then cooled potatoes, unripe bananas and apples) are incorporated with the goal of increased short chain fatty acid production and to promote microbiome homeostasis. Phase 2 (Weeks 7–12) reduces PEN component to 25% and allows for additional fruits/vegetables. Phase 3 (maintenance) removes mandatory foods and allows one or two homemade “free” meals while continuing to avoid ultra‐processed foods. Real‐world descriptions of the CDED are limited^22^, ^23^, ^24^ and, to date, no North American center has shared their real‐world experience. Here, we offer analysis of the CDED in practice at a tertiary care children's hospital in the United States.

## METHODS

### Study population

This is a retrospective cohort study of pediatric patients (≤18 years) with CD who received dietary education regarding the CDED at our referral center from January 2020 through December 31, 2023. Within our inflammatory bowel disease (IBD) center, all patients with a diagnosis of CD based on clinical, endoscopic, and histologic findings were included in the initial review; patients initiating the CDED were identified by departmental lists. At our center, consensus practice among IBD providers is to recommend an IBD‐focused dietitian visit for all patients at diagnosis and for those considering changes in therapy. Patients meeting with a dietitian but not adopting the CDED received in‐person education regarding general dietary considerations and were provided with written materials. For patients initiating the CDED, the standard practice was an index in‐person visit with an IBD‐focused dietitian, followed by a 4‐ to 6‐week return visit and interval telephone and electronic‐medical record messaging, along with a 3‐month follow‐up with their primary IBD provider. Written materials and CDED‐focused websites were provided as part of dietary education. Patients were counseled to pursue PEN with Modulen (if accessible) or similar oral nutrition products with sufficient calories and an appropriate balance of macronutrients, noting that low calorie, high protein options were not suitable alternatives and may lead to malnutrition with micronutrient deficiencies. All patients adopting the CDED were included in end‐point analysis. This study was approved by the institutional review board at the Ann & Robert H. Lurie Children's Hospital of Chicago.

### Data collection

All data was collected via review of electronic medical records. Information was gathered regarding demographics, disease classification (Montreal), previous medical therapy exposure, concomitant therapies, CDED initiation date and length of therapy. Disease activity was measured via the Pediatric Crohn's Disease Activity Index (PCDAI). Laboratory results (hemoglobin, albumin) and biomarker data (C‐reactive protein (CRP), calprotectin) were gathered before intervention and at follow‐up. Data on side effects were collected as reported through last follow‐up. Simple‐endoscopic scoring for CD was used for mucosal response and remission.

### Statistical approach and outcomes

The primary outcome was measured as steroid‐free clinical remission (SR‐CR) at Weeks 6, 12, and 24 defined as a PCDAI of 10 or less without concomitant corticosteroid use. Endoscopic response was defined as a decrease of **≥**50% in the simple endoscopic score for Crohn's disease (SES‐CD) score from baseline and endoscopic remission as a SES‐CD ≤2. Patient characteristics were summarized using frequency (*n*) and percentage (%) for categorical variables, mean ± standard deviation for normally distributed continuous variables, and median (25th percentile, 75th percentile) for non‐normally distributed continuous variables. The Shapiro‐Wilks test was used to evaluate the normality of continuous data. A Friedman test was conducted on PCDAI scores to examine the effect of the CDED over four time points (i.e., the last encounter prior to CDED initiation, Weeks 6, 12, and 24). A Wilcoxon signed‐rank test was used to compare calprotectin levels and CRP levels before and after the CDED, respectively.

## RESULTS

### Patient characteristics

A total of 419 patients with CD were seen during the study window, of whom 32 subjects (7.6%) received counseling on the CDED from an IBD‐focused dietitian. Of these patients, 24 (75%) went on to initiate the CDED (Figure S1). In subjects adopting the diet, 6 (25%) were female and most, 20 (83%), self‐identified as Caucasian. The mean age of diagnosis was 11.0 years (SD = 3.6). Most patients received focused CDED counseling early in their disease course (median 2.4 months after diagnosis, range 0.9, 9.2).

Three quarters of subjects had ileocolonic CD and 6 (25%) had upper gastrointestinal involvement. Based on initial activity index and Montreal classification, disease burden in this cohort was mild; 23 (96%) were classified as non‐stricturing/non‐penetrating and only 3 (13%) had a history of any IBD‐related surgery. The majority of patients (54%) had prior/concomitant exposure to immunomodulator or advanced therapy, though 17 (70.8%) initiated CDED as monotherapy. Induction of the CDED overlapped with corticosteroid usage in 11 (46%) subjects (Table 1).

**Table 1:** Subject demographics and clinical characteristics.

	Subjects on the CDED, *N* = 24
**Sex,** ***n*** **(%)**	
Male	18 (75)
Female	6 (25)
**Race,** ***n*** **(%)**	
Asian or Pacific Islander	1 (4)
White	20 (83)
Other or not reported	3 (13)
**Ethnicity,** ***n*** **(%)**	
Hispanic	2 (8)
Non‐Hispanic	21 (88)
Not reported	1 (4)
**Age at diagnosis (years), mean (** ***SD*** **)**	11.0 (3.6)
**Months from diagnosis to CDED, median (IQR)**	2.4 (0.9, 9.2)
**Length of follow‐up on CDED (months), median (IQR)**	23.6 (14.3, 37.1)
**Family history,** ***n*** **(%)**	
Yes	9 (38)
No	1 (4)
Not reported	14 (58)
**Montreal Disease Location/Behavior,** ***n*** **(%)**	
L1: terminal ileum	2 (8)
L2: colon	3 (13)
L3: ileocolonic	13 (54)
L3 + L4: ileocolonic + upper GI	5 (21)
L1 + L4: terminal ileum + upper GI	1 (4)
B1: non‐stricturing, non‐penetrating	23 (96)
B2: structuring	1 (4)
**History of IBD‐related surgery**	
Yes	3 (13)
No	21 (88)
**Prior exposure to therapy,** ***n*** **(%)**	
None	11 (46)
Methotrexate	4 (16)
6‐MP/azathioprine	4 (16)
Infliximab	5 (21)
Adalimumab	4 (16)
Ustekinumab	3 (13)
**Steroid with CDED induction**	
Prednisone	10 (42)
Budesonide	3 (13)
**Concomitant therapy with CDED** ***n*** **(%)**	
Yes	7 (29)
Methotrexate	3 (13)
Ustekinumab	4 (17)
Adalimumab	1 (4)
Vedolizumab	1 (4)
None (monotherapy)	18 (75)
**Patient‐reported side effects**	
Diarrhea/urgency	4 (16)
Abdominal discomfort with some allowed foods	6 (25)
Weight loss	2 (8)
None	15 (63)

*Note*: Data were reported as *n* (%) for categorical variables, mean (*SD*) for normally distributed variable, and median (IQR) for non‐normally distributed variables.

Abbreviations: 6‐MP, 6‐mercaptopurine; CDED, Crohn's Disease Exclusion Diet; IBD, inflammatory bowel disease.

### Diet adherence and clinical remission at Weeks 6, 12, and 24

**Figure 1:**
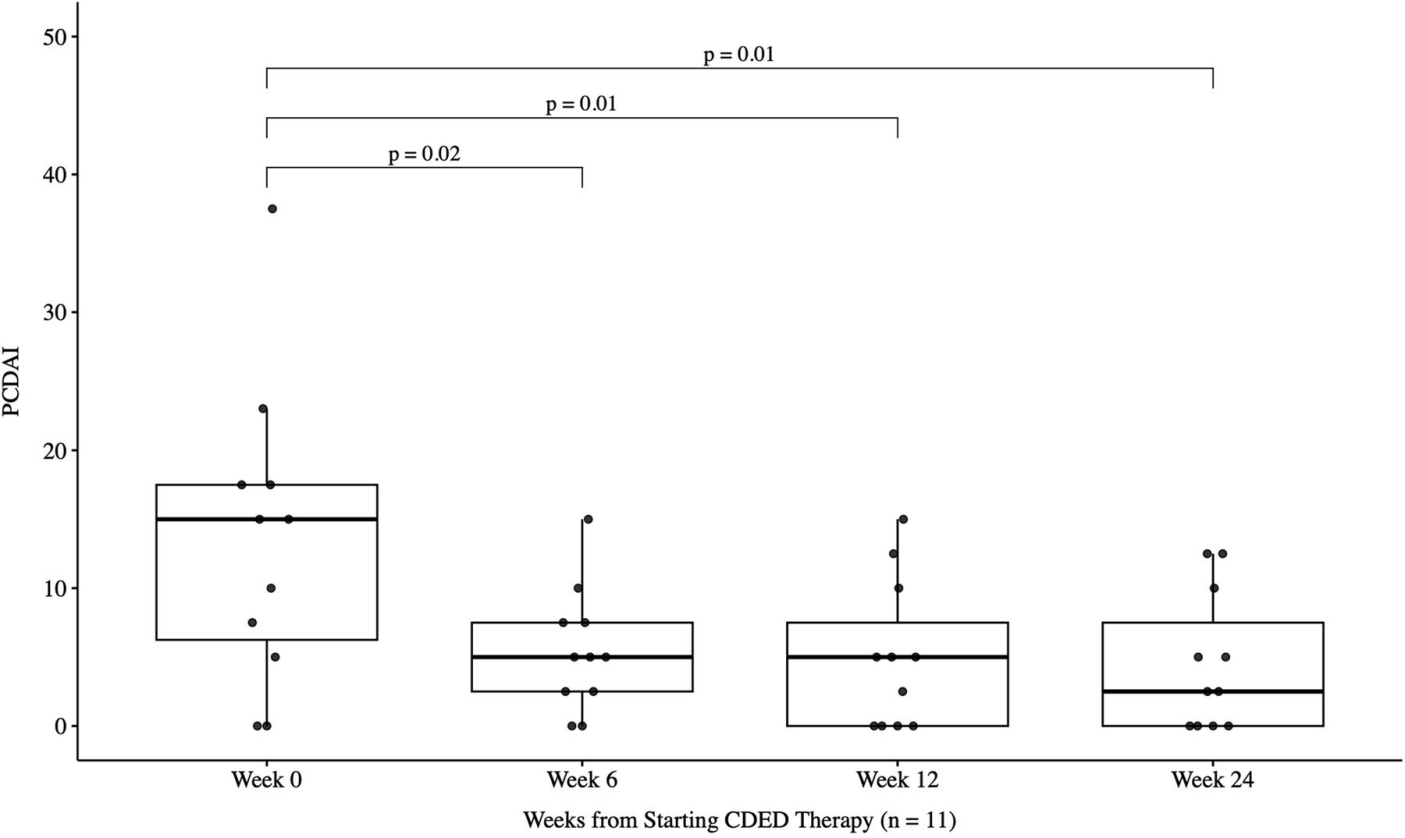
Changes in PCDAI from Week 0 to Weeks 6, 12, and 24, *n* = 11. In examining the effects of CDED therapy over time, a Friedman test was conducted on the PCDAI scores. The analysis revealed a statistically significant difference in PCDAI scores across the time points, *χ* ^2^(3) = 13.13, *P* = 0.004. Following the significant Friedman test result, post hoc analysis using the Wilcoxon test was performed to assess pairwise differences between the time points. The analysis indicated significant differences between Weeks 6, 12, and 24 compared with baseline (all *P* < 0.05).

All 24 subjects initiating the CDED were included in the assessment of clinical remission. The median PCDAI values at Weeks 0 and 24 were 12.5 (IQR, 1.9–16.9) and 2.5 (IQR, 0–8.8), respectively. Compared with Week 0, PCDAI scores were significantly lower at Weeks 6, 12, and 24 (*p* = 0.05) (Figure 1).

**Figure 2:**
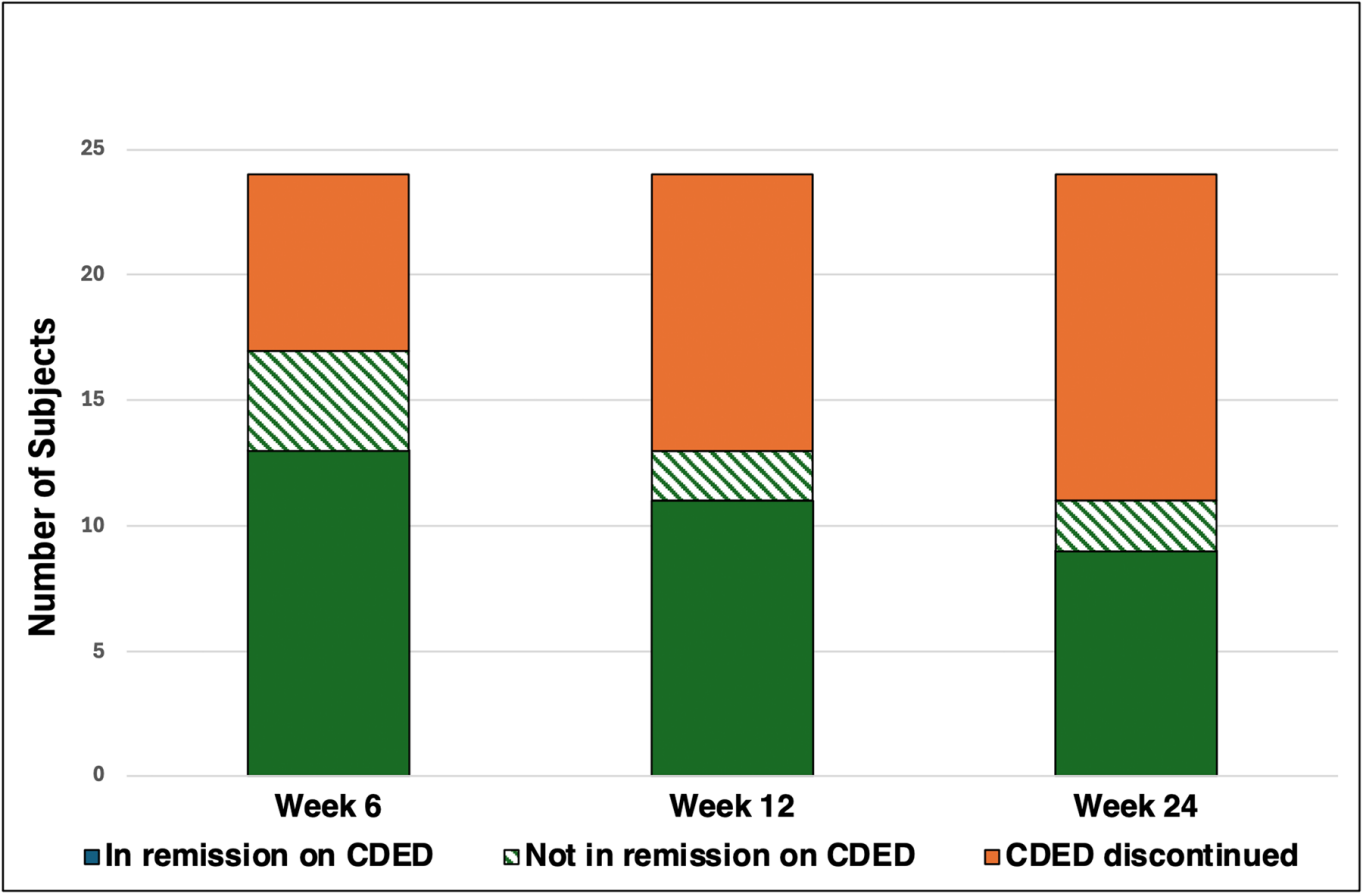
Clinical remission at Weeks 6, 12, and 24 for all subjects starting the CDED.

At Weeks 6, 12, and 24, the diet was continued in 70.1%, 54.2%, and 45.8% of subjects, respectively. While nine (37.5%) subjects from the initial cohort met criteria for corticosteroid‐free clinical remission (SF‐CR) at Week 24, in those that maintained the CDED, rates of SF‐CR were 13/17 (76.5%) at Week 6, 11/13 (84.6%) at Week 12, and 9/11 (81.8%) at Week 24 (Figure 2). Of the 11 subjects on the CDED at Week 24, 5 subjects began CDED with a baseline PDCAI ≤10 and maintained remission over the course of the intervention (Table 2).

**Table 2:** Individual subject clinical data.

Patient ID	Diagnosis to CDED (days)	Gender, age [years]	Disease location (montreal)	Prior therapy	Concomitant therapy	Corticosteroid induction (week discontinued)	Duration CDED + PEN (week #)	Phase of CDED reached	Patient‐reported side effects	Calprotectin pre/post CDED	PCDAI Week 0	PCDAI Week 6	PCDAI Week 12	PCDAI Week 24	SES‐CD pre/post CDED (week on diet)
1	28	M, [13]	L3 + L4			No	83	Phase 3		2370/6	37.5	7.5	5	0	15/0 (Week 75)
2	216	M, [16]	L3 + L4			No	51	Phase 3	Weight loss	2460/382	15	10	10	5	9/6 (Week 49)
3	27	M, [12]	L3			Yes (6)	146	Phase 1^a^	Abdominal discomfort, diarrhea	Data unavailable	23	7.5	12.5	12.5	21/3 (Week 49)
4	81	M, [17]	L3			No	143	Phase 3		541/35	0	0	0	0	0/0 (Week 134)
5	1624	M, [13]	L3	IFX	MTX, UST	No	192	Phase 3	Weight loss	1230/429	0	0	0	2.5	8/4 (Week 50)
6	40	M, [8]	L3			Yes (3)	69	Phase 3		5380/2000	7.5	2.5	2.5	2.5	12/12 (Week 60)
7	138	M, [9]	L1			No	24	Phase 3		526/123	5	5	5	5	
8	9	M, [11]	L1			No	63	Phase 3		2000/862	15	15	5	10	
9	10	F, [13]	L3	MTX		No	54	Phase 3	Abdominal discomfort	1340/40	10	5	0	0	13/4 (Week 50)
10	56	M, [14]	L3			No	30	Phase 3		7840/80	17.5	5	0	0	
11	484	M, [13]	L1 + L4		MTX	No	27	Phase 3		1090/274	17.5	2.5	15	12.5	
															Therapy discontinuation
12	65	M, [8]	L3 + L4			Yes (12)	12	Phase 2		266/774	5	5	5	NA	Lack of clinical response
13	92	F, [9]	L2	5‐ASA		Yes (4)	12	Phase 2	Diarrhea	381/420	15	5	0	NA	Difficulty with adherence
14	61	F, [15]	L3			Yes (6)	11	Phase 1^b^		1820/183	25	20	NA	NA	Diet unpalatable
15	209	F, [14]	L3	MTX, 6MP, ADA		Yes (2)	11	Phase 1		82/529	10	5	NA	NA	Lack of clinical response
16	956	M, [15]	L3	IFX, ADA	VEDO	Yes^c^	6	Phase 1		4430/3100	32.5	27.5	NA	NA	Lack of clinical response
17	132	F, [14]	L3		ADA	No	6	Phase 1	Abdominal discomfort	1290/8000	10	25	NA	NA	Lack of clinical response, diet unpalatable, side effects
18	29	M, [9]	L3	IFX		No	6	Phase 1		5260/119	15	NA	NA	NA	Diet unpalatable
19	694	M, [15]	L2	IFX	UST	No	3	Phase 1	Abdominal discomfort, diarrhea	Data unavailable	10	NA	NA	NA	Lack of clinical response, side effects
20	4078	M, [13]	L2	5‐ASA, MTX, IFX, ADA	MTX, UST, Metronidazole	Yes^c^	3	Phase 1	Abdominal discomfort	1450/3440	17.5	NA	NA	NA	Lack of clinical response, diet unpalatable, side effects
21	33	M, [11]	L3			Yes (6)	2	Phase 1		2850/220	10	NA	NA	NA	Lack of clinical response
22	8	F, [13]	L3			No	2	Phase 1		Data unavailable	17	NA	NA	NA	Diet unpalatable
23	2211	M, [13]	L3 + L4	6MP, ADA, MTX	UST	Yes^c^	1	Phase 1		>800/>800	27.5	NA	NA	NA	Unknown
24	25	M, [8]	L3 + L4			No	1	Phase 1	Abdominal discomfort, diarrhea	Data unavailable	32.5	NA	NA	NA	Side effects

*Note*: Solid line denotes the 24‐week cohort (above) vs. those discontinuing CDED sooner (below).

Abbreviations: 6MP, 6‐mercaptopurine; ADA, adalimumab; CDED, Crohn's Disease Exclusion Diet; F, female; IFX, infliximab; M, male; MTX, methotrexate; PCDAI, pediatric Crohn's disease activity index; SES‐CD, simple endoscopic scoring; UST, ustekinumab.

^a^ Fluctuated between Phases 1–3 for 1 year; reintroducing foods led to abdominal discomfort.

^b^ Maintained Phase 1 for 11 weeks.

^c^ Remained on corticosteroid at last follow‐up.

Reasons for discontinuing the diet included lack of perceived clinical improvement (53.8%), difficulty with diet adherence/unpalatability (46.2%), and patient reported side effects (30.8%) (Table 2). Following discontinuation of the CDED, seven patients started anti‐TNF (infliximab or adalimumab), three ustekinumab, one vedolizumab, one mesalamine, and another had a diverting ileostomy. Of these, four subjects required a course of bridging corticosteroid.

### Week 24 cohort analysis

There were 11 subjects that remained on the diet at Week 24, with a median follow‐up of 21.4 months (IQR,14.8, 38.2). Of these, only one subject was female (9.1%) compared with 5/13 (38.5%) in the cohort discontinuing the diet. Only two subjects received concomitant corticosteroid induction and, in both cases, it had been discontinued by Week 6 (compared with 8/13 subjects stopping the diet before Week 24). The CDED was used as monotherapy in nine of these patients (81.8%) vs. 8/13 (61.5%) in those discontinuing the diet. Two subjects used the diet adjunctively continuing previous medical therapy. Disease location and disease severity at the start of CDED were similar when comparing each cohort (Table 3).

**Table 3:** Subjects maintaining vs. discontinuing the CDED.

Subject demographics	<24 weeks (*n* = 13)	24 weeks or more (*n* = 11)	*P*‐Value
**Sex,** ***n*** **(%)**			0.17
Female	5 (38)	1 (9)	
Male	8 (62)	10 (91)	
**Age at diagnosis (years), mean (SD)**	12.08 (2.72)	12.64 (2.66)	0.88
**Disease location,** ***n*** **(%)**			0.22
L1: terminal ileum	0	2 (18)	
L2: colon	3 (23)	0	
L3: ileocolonic	7 (54)	6 (55)	
L3 + L4: ileocolonic + upper GI	3 (23)	2 (18)	
L1 + L4: terminal ileum + upper GI	0	1 (9)	
**Disease behavior,** ***n*** **(%)**			1
B1: non‐stricturing, non‐penetrating	12 (92)	11 (100)	
B2: stricturing	1 (8)	0	
**Days from diagnosis to starting CDED, median (IQR)**	92 (33, 694)	56 (28, 177)	0.42
**Prior therapy,** ***n*** **(%)**			0.1
Yes	7 (54)	2 (18)	
5‐ASA	2	0	
Methotrexate	3	1	
6‐MP/azathioprine	2	0	
Infliximab	4	1	
Adalimumab	4	0	
Ustekinumab	0	0	
**Concomitant therapy,** ***n*** **(%)**			0.39
Yes	5 (38)	2 (18)	
Methotrexate	1	2	
Ustekinumab	2	1	
Adalimumab	1	0	
Vedolizumab	1	0	
**PCDAI baseline, median (IQR)**	15 (10, 25)	15 (6, 18)	0.37
**Calprotectin baseline, median (IQR)**	1370 (486, 2593)	1670 (1125, 2438)	0.47

*Note*: Continuous variables were compared between groups using the Wilcoxon rank‐sum test, and categorical variables were compared using Fisher's exact test.

Abbreviation: 6‐MP, 6‐mercaptopurine.

### Biomarker response and mucosal healing

A significant decrease in calprotectin levels was observed in those reaching Week 24 compared with baseline (*p* < 0.01, 95% CI for the median of the pairwise differences: 504.0, 3983.2). See Table 2 for further detail. Other indices, including CRP, albumin, and hemoglobin, did not have statistically significant differences between Weeks 0 and 24.

Within this cohort, 7/11 (63.6%) patients underwent endoscopic assessment after at least 24 weeks of the CDED. The median SES‐CD prior to adopting the diet was 9 (IQR, 8.4, 12.5) and at follow‐up was measured at 4 (IQR, 1.3, 5). Endoscopic response was noted in 5/7 (71.4%) cases, with two subjects found to be in endoscopic remission (28.6%).

### Side effects

Side effects reported to be associated with the CDED were mild and transient, occurring in nine (37.5%) subjects, with improvement over time in those maintaining adherence to the diet. This included abdominal discomfort with initiation of the diet in six subjects (25%), loose stools/urgency in four (16.7%), and weight loss in two (8.3%). In the two subjects experiencing weight loss, one subject lost 3% of their body weight and the other 7.5% of their body weight. In each case, weight trends stabilized moving from phase 1 to phase 2 of the diet. Adoption of the diet was tolerated with no reported side effects in 15/24 (62.5%) subjects.

## DISCUSSION

Here, we describe successful implementation of the CDED in a select population of children and adolescents with mild‐moderate CD. To our knowledge, this is the first single‐center North American experience sharing outcomes of the CDED. Though the numbers are small we demonstrate that, for many, the diet is sustainable. Additionally, for those maintaining the diet at Week 24, clinical effectiveness is clear, with improvement in activity scoring and stool calprotectin.

In our cohort, high remission rates were noted in those adhering to the diet at Weeks 6 and 12 (76.5% and 84.6%, respectively). This experience mirrors previous descriptions of the CDED, which has largely focused on induction of remission.

Week 6 response with the CDED was initially detailed in a cohort of 47 pediatric subjects, with 30 (70.2%) meeting criteria for remission,^25^ and subsequently described in a cohort of children and young adults (*n* = 21) where disease had been refractory to advanced therapies, with 62% achieving clinical remission.^26^ More recently, these results have been reproduced in a pediatric experience of CDED + PEN induction, where formula type and structure did not impact rates of remission, 17/24 (70.8%) at Week 6.^27^

Beyond Week 6 assessments, the seminal randomized‐controlled trial of the CDED + PEN found that 28/37 (75.6%) subjects were in SF‐CR remission at Week 12.^28^ Likewise, in two retrospective cohorts, 46/66 (69.7%) met criteria for clinical remission after 8 weeks^22^ and 30/48 (62.5%) achieved remission at Week 12.^29^

In our assessment of induction with the CDED, when including those who had discontinued therapy due to intolerance, side effects, or lack of clinical improvement, remission rates dropped to 54.2% and 45.8% by Weeks 6 and 12. Even as the CDED purports an advantage with dietary tolerance compared with EEN,^28^ our numbers reflect a more modest real‐world efficacy. In our experience, difficulty with adherence/unpalatability accounted for discontinuation in less than half of cases, with lack of clinical response being the most common driver of diet cessation. Concomitant corticosteroid was more common in the cohort discontinuing therapy before Week 24, raising the possibility that disease activity and diet tolerance may be inversely related.

These findings are similar to outcomes reported from an open‐label randomized trial of adult patients receiving the CDED with or without PEN.^20^ In this cohort of bio‐naive patients (*n* = 40), 62.5% were in clinical remission at Week 6, and by Week 12, 35% had discontinued therapy or became poorly compliant. Similar to our data, in this cohort, 81.8% of those maintaining the diet at Week 6 remained in remission at Week 24.

We note that, in subjects persisting on the CDED until at least Week 24, 9/11 achieved clinical remission. This finding was bolstered by a statistically significant drop in fecal calprotectin from Weeks 0 to 24, with 50% of subjects having complete normalization (<200 µg/g). Similar changes in calprotectin have been described in a cohort of children on the CDED (median value of 1045.00 µg/g, IQR = 1188.00 at Week 0 and median value of 363.00 µg/g, IQR = 665.00 at Week 12 (*p* < 0.05)).^30^ Though differences between subjects discontinuing therapy before and after Week 24 were not statistically significant, our analysis suggests that, for those earlier in the diagnosis, with less prior medication exposure, and not requiring concomitant corticosteroid, success may be more likely.

Though the numbers are small, our study is also the first to report on endoscopic response following a sustained course of the CDED in the pediatric population. We described mucosal response in 5/7 (71.4%) patients undergoing repeat endoscopic assessment at a minimum of 24 weeks. Two subjects achieved mucosal healing (SES‐CD = 0). This was similar to endoscopic response rates (84.6%, *n* = 13) reported after a modified CDED with PEN, noting that repeat assessment came after just 6 weeks of dietary therapy in this cohort.^31^ In an adult population on the CDED with or without PEN, 14/40 (35%) met criteria for endoscopic remission (defined as SES‐CD ≤3) at Week 24.^20^

Side effects were mild and included increased abdominal discomfort, urgency, loose stools, and weight loss. For the latter, downtrends in weight were slight and transient. While this is a common concern with application of dietary therapy, preliminary data suggests that the CDED may confer superior weight gain compared with induction with EEN.^32^, ^33^ However, even when side effects were not severe, the CDED may have impacted quality of life and diminished diet persistence. Assessment of side effects is limited by documentation at the time of follow‐up visits and determinations regarding the effect of the CDED on quality of life, eating behaviors, body image concerns, or financial constraints, were not assessed as part of this study.

Strengths of this study include reporting of outcomes beyond Week 24 with a median length of follow‐up of nearly 2 years for subjects maintaining the diet, along with endoscopic reassessment in 63.6% of these subjects. Determining long‐term sustainability of the CDED will be critical moving forward. Encouragingly, prospective dietary assessments beyond Week 52 in a cohort of patients following the CDED suggests a lasting impact with improved dietary habits and decreased exposure to ultra processed foods.^34^

Limitations of this study, and others of its kind, include the retrospective focus, sample size, and inability to track adherence to the CDED apart from reporting at the time of scheduled follow‐up. While less than half of the patients in our cohort were induced with concomitant corticosteroid, this may confound the assessment of response. Notably, in the Week 24 cohort, only two patients received induction corticosteroid, and one of those subjects did not achieve SF‐CR. Likewise, only two patients in this cohort were on concomitant therapy (ustekinumab, methotrexate) and the CDED was used adjunctively to achieve remission. In a population of patients with mild disease and no perianal findings, the PCDAI may limit assessment of clinical response. While our results underscore the underutilization of dietary therapy in the United States,^35^ interpretation is limited, as reporting on the number of subjects that may have been poor candidates for dietary therapy was not available.

We note that generalizability is a potential limitation of this study. Our population included a group of highly‐motivated patients and families with the resources needed to procure the dietary staples of the CDED. It will be imperative moving forward for all dietary therapies to offer customizability respecting cultural and community traditions if uptake is to be improved. Dietitian education and support will be critical in this effort.^32^

Looking to the future, while dietary therapy has traditionally been supported as effective for mild‐moderate ileal disease, innovative research efforts have examined outcomes in populations with colitis. This includes a randomized controlled trial examining EEN use in patients hospitalized with acute severe colitis^36^ and pilot data characterizing outcomes of an exclusion diet for patients with ulcerative colitis.^37^ This work underscores the need for prospective trials of the CDED in children with diverse IBD phenotypes and of various socioeconomic backgrounds. Given that success of dietary therapy is closely linked to adherence, careful assessment of individual diet intake across the CDED phases will be critical.

Along with a need for greater clarity on response with disease location, our work highlights the investment required for dietitian support in IBD care. While few patients had met with an expert dietitian on the CDED, in those that did, uptake was high (75%). Ample evidence exists which demonstrates the unwavering interest of patients and families in dietary approaches to IBD management.^38^, ^39^, ^40^ As the American Gastroenterological Association has recently published a clinical practice update outlining recommendations for dietary therapy in IBD,^19^, ^40^ more must be done to enable successful implementation.

## CONCLUSION

For patients with CD who met with an IBD‐focused dietitian to discuss the CDED, rates of uptake were high, even as adherence remains a challenge. Our real‐world experience from a tertiary care center in North America suggests that, in a population of children with mild disease activity, the CDED may offer a safe and effective means of achieving and maintaining remission.

## AUTHOR CONTRIBUTIONS

*Study concept and design*: Joseph Runde, Mayah Greenfield, and Caroline Salozzo. *Acquisition of data*: Joseph Runde, Mayah Greenfield, and Wenya Chen. *Analysis and interpretation of data*: Joseph Runde, Mayah Greenfield, and Wenya Chen. *Drafting of manuscript*: Joseph Runde, Mayah Greenfield, Wenya Chen, Jennifer Strople, and Jeffrey Brown. *Critical revision of manuscript*: Joseph Runde, Mayah Greenfield, and Wenya Chen.

## CONFLICT OF INTEREST STATEMENT

None declared.

## Supporting information

Figure S1: Flow Diagram of Study Subjects. Of note, in those subjects that did not meet with an IBD‐focused dietitian eligibility for the CDED was not assessed.

## References

[1] Debruyn JCC , Soon IS , Hubbard J , Wrobel I , Panaccione R , Kaplan GG . Nationwide temporal trends in incidence of hospitalization and surgical intestinal resection in pediatric inflammatory bowel diseases in the United States from 1997 to 2009. Inflamm Bowel Dis. 2013;19(11):2423‐2432. 23974991 10.1097/MIB.0b013e3182a56148

[2] Murthy SK , Begum J , Benchimol EI , et al. Introduction of anti‐TNF therapy has not yielded expected declines in hospitalisation and intestinal resection rates in inflammatory bowel diseases: A population‐based interrupted time series study. Gut. 2020;69(2):274‐282. 31196874 10.1136/gutjnl-2019-318440 PMC6984056

[3] Coward S , Benchimol EI , Bernstein CN , et al. Forecasting the incidence and prevalence of inflammatory bowel disease: A Canadian nationwide analysis. Am J Gastroenterol. 2024;119(8):1563‐1570. 38299598 10.14309/ajg.0000000000002687 PMC11288393

[4] Kuenzig ME , Fung SG , Marderfeld L , et al. Twenty‐first century trends in the global epidemiology of pediatric‐onset inflammatory bowel disease: Systematic review. Gastroenterology. 2022;162(4):1147‐1159.e4. 34995526 10.1053/j.gastro.2021.12.282

[5] Herzog D , Fournier N , Buehr P , et al. Prevalence of intestinal complications in inflammatory bowel disease: A comparison between paediatric‐onset and adult‐onset patients. Eur J Gastroenterol Hepatol. 2017;29(8):926‐931. 28471820 10.1097/MEG.0000000000000896

[6] Jakobsen C , Bartek J , Wewer V , et al. Differences in phenotype and disease course in adult and paediatric inflammatory bowel disease—A population‐based study. Aliment Pharmacol Ther. 2011;34(10):1217‐1224. 21981762 10.1111/j.1365-2036.2011.04857.x

[7] Olén O , Askling J , Sachs MC , et al. Increased mortality of patients with childhood‐onset inflammatory bowel diseases, compared with the general population. Gastroenterology. 2019;156(3):614‐622. 30342031 10.1053/j.gastro.2018.10.028

[8] Kotlyar DS , Osterman MT , Diamond RH , et al. A systematic review of factors that contribute to hepatosplenic T‐cell lymphoma in patients with inflammatory bowel disease. Clin Gastroenterol Hepatol. 2011;9(1):36‐41.e1. 20888436 10.1016/j.cgh.2010.09.016

[9] Massano A , Bertin L , Zingone F , et al. Extraintestinal cancers in inflammatory bowel disease: A literature review. Cancers. 2023;15(15):3824. 10.3390/cancers15153824 37568640 PMC10417189

[10] Svolos V , Gerasimidis K , Buchanan E , et al. Dietary treatment of Crohn's disease: Perceptions of families with children treated by exclusive enteral nutrition, a questionnaire survey. BMC Gastroenterol. 2017;17(1):14. 10.1186/s12876-016-0564-7 28103809 PMC5247812

[11] Jatkowska A , White B , Jaskolski P , et al. Perceptions toward established and novel dietary therapies for Crohn's disease management among adult patients: Results from a questionnaire survey. Crohns Colitis 360. 2024;6(1):otae008. 38464347 10.1093/crocol/otae008 PMC10924435

[12] Mehta P , Pan Z , Furuta GT , Kim DY , de Zoeten EF . Parent perspectives on exclusive enteral nutrition for the treatment of pediatric Crohn disease. J Pediatr Gastroenterol Nutr. 2020;71(6):744‐748. 32910623 10.1097/MPG.0000000000002847

[13] Narula N , Dhillon A , Zhang D , Sherlock ME , Tondeur M , Zachos M . Enteral nutritional therapy for induction of remission in Crohn's disease. Cochrane Database Syst Rev. Published online 2018;2018(4): CD000542. 10.1002/14651858.cd000542.pub3 PMC6494406 29607496

[14] Buchanan E , Gaunt WW , Cardigan T , Garrick V , McGrogan P , Russell RK . The use of exclusive enteral nutrition for induction of remission in children with Crohn's disease demonstrates that disease phenotype does not influence clinical remission. Aliment Pharmacol Ther. 2009;30(5):501‐507. 19549288 10.1111/j.1365-2036.2009.04067.x

[15] Borrelli O , Cordischi L , Cirulli M , et al. Polymeric diet alone versus corticosteroids in the treatment of active pediatric Crohn's disease: A randomized controlled open‐label trial. Clin Gastroenterol Hepatol. 2006;4(6):744‐753. 10.1016/j.cgh.2006.03.010 16682258

[16] Grover Z , Burgess C , Muir R , Reilly C , Lewindon PJ . Early mucosal healing with exclusive enteral nutrition is associated with improved outcomes in newly diagnosed children with luminal Crohn's disease. J Crohns Colitis. 2016;10(10):1159‐1164. 26980840 10.1093/ecco-jcc/jjw075

[17] Pigneur B , Lepage P , Mondot S , et al. Mucosal healing and bacterial composition in response to enteral nutrition vs steroid‐based induction therapy—A randomised prospective clinical trial in children with Crohn's disease. J Crohns Colitis. 2019;13(7):846‐855. 30541015 10.1093/ecco-jcc/jjy207

[18] Critch J , Day AS , Otley A , et al. Use of enteral nutrition for the control of intestinal inflammation in pediatric Crohn disease. J Pediatr Gastroenterol Nutr. 2012;54(2):298‐305. 22002478 10.1097/MPG.0b013e318235b397

[19] Hashash JG , Elkins J , Lewis JD , Binion DG . AGA clinical practice update on diet and nutritional therapies in patients with inflammatory bowel disease: Expert review. Gastroenterology. 2024;166(3):521‐532. 38276922 10.1053/j.gastro.2023.11.303

[20] Yanai H , Levine A , Hirsch A , et al. The Crohn's disease exclusion diet for induction and maintenance of remission in adults with mild‐to‐moderate Crohn's disease (CDED‐AD): An open‐label, pilot, randomised trial. Lancet Gastroenterol Hepatol. 2022;7(1):49‐59. 34739863 10.1016/S2468-1253(21)00299-5

[21] Sigall Boneh R , Westoby C , Oseran I , et al. The Crohn's disease exclusion diet: A comprehensive review of evidence, implementation strategies, practical guidance, and future directions. Inflamm Bowel Dis. Published online November 18 2023;30(10):1888. 10.1093/ibd/izad255 PMC11446999 37978895

[22] Scarallo L , Banci E , De Blasi A , et al. A real‐life pediatric experience of Crohn's disease exclusion diet at disease onset and in refractory patients. J Pediatr Gastroenterol Nutr. 2024;79(3):592‐601. 38962891 10.1002/jpn3.12283

[23] Correia I , Oliveira P , Antunes M , Raimundo M , Moreira A . Is there evidence of Crohn's Disease Exclusion Diet (CDED) in remission of active disease in children and adults? A systematic review. Nutrients. 2024;16(7):987. 10.3390/nu16070987 38613020 PMC11013840

[24] Jijón Andrade MC , Pujol Muncunill G , Lozano Ruf A , et al. Efficacy of Crohn's disease exclusion diet in treatment ‐naïve children and children progressed on biological therapy: A retrospective chart review. BMC Gastroenterol. 2023;23(1):225. 37386458 10.1186/s12876-023-02857-6 PMC10311743

[25] Sigall‐Boneh R , Pfeffer‐Gik T , Segal I , Zangen T , Boaz M , Levine A . Partial enteral nutrition with a Crohn's disease exclusion diet is effective for induction of remission in children and young adults with Crohn's disease. Inflamm Bowel Dis. 2014;20(8):1353‐1360. 24983973 10.1097/MIB.0000000000000110

[26] Sigall Boneh R , Sarbagili Shabat C , Yanai H , et al. Dietary therapy with the Crohn's disease exclusion diet is a successful strategy for induction of remission in children and adults failing biological therapy. J Crohns Colitis. 2017;11(10):1205‐1212. 28525622 10.1093/ecco-jcc/jjx071

[27] Landorf E , Hammond P , Abu‐Assi R , et al. Formula modifications to the Crohn's disease exclusion diet do not impact therapy success in paediatric Crohn's disease. J Pediatr Gastroenterol Nutr. 2024;78(6):1279‐1286. 38623960 10.1002/jpn3.12215

[28] Levine A , Wine E , Assa A , et al. Crohn's disease exclusion diet plus partial enteral nutrition induces sustained remission in a randomized controlled trial. Gastroenterology. 2019;157(2):440‐450.e8. 31170412 10.1053/j.gastro.2019.04.021

[29] Fliss‐Isakov N , Aviv Cohen N , Bromberg A , et al. Crohn's disease exclusion diet for the treatment of Crohn's disease: Real‐world experience from a tertiary center. J Clin Med. 2023;12(16):5428. 10.3390/jcm12165428 37629470 PMC10455757

[30] Matuszczyk M , Meglicka M , Wiernicka A , et al. Effect of the Crohn's Disease Exclusion Diet (CDED) on the fecal calprotectin level in children with active Crohn's disease. J Clin Med. 2022;11(14):4146. 10.3390/jcm11144146 35887910 PMC9317017

[31] Urlep D , Orel R , Kunstek P , Benedik E . Treatment of active Crohn's disease in children using partial enteral nutrition combined with a modified Crohn's disease exclusion diet: A pilot prospective cohort trial on clinical and endoscopic outcomes. Nutrients. 2023;15(21):4676. 10.3390/nu15214676 37960328 PMC10650058

[32] Sigall Boneh R , Park S , Soledad Arcucci M , et al. Cultural perspectives on the efficacy and adoption of the Crohn's disease exclusion diet across diverse ethnicities: A case‐based overview. Nutrients. 2024;16(18):3184. 10.3390/nu16183184 39339784 PMC11434781

[33] Niseteo T , Sila S , Trivić I , Mišak Z , Kolaček S , Hojsak I . Modified Crohn's disease exclusion diet is equally effective as exclusive enteral nutrition: Real‐world data. Nutr Clin Pract. 2022;37(2):435‐441. 34339527 10.1002/ncp.10752

[34] Martín‐Masot R , Herrador‐López M , Navas‐López VM . Dietary habit modifications in paediatric patients after one year of treatment with the Crohn's disease exclusion diet. Nutrients. 2023;15(3):554. 10.3390/nu15030554 36771261 PMC9921286

[35] Lawley M , Wu JW , Navas‐López VM , et al. Global variation in use of enteral nutrition for pediatric Crohn disease. J Pediatr Gastroenterol Nutr. 2018;67(2):e22‐e29. 10.1097/MPG.0000000000001946 29543696

[36] Sahu P , Kedia S , Vuyyuru SK , et al. Randomised clinical trial: Exclusive enteral nutrition versus standard of care for acute severe ulcerative colitis. Aliment Pharmacol Ther. 2021;53(5):568‐576. 33440046 10.1111/apt.16249

[37] Sarbagili‐Shabat C , Albenberg L , Van Limbergen J , et al. A novel UC Exclusion diet and antibiotics for treatment of mild to moderate pediatric ulcerative colitis: A prospective open‐label pilot study. Nutrients. 2021;13(11):3736. 10.3390/nu13113736 34835992 PMC8622458

[38] De Vries JHM , Dijkhuizen M , Tap P , Witteman BJM . Patient's dietary beliefs and behaviours in inflammatory bowel disease. Dig Dis. 2019;37(2):131‐139. 30391940 10.1159/000494022 PMC6381876

[39] Chuong KH , Haw J , Stintzi A , Mack DR , O'Doherty KC . Dietary strategies and food practices of pediatric patients, and their parents, living with inflammatory bowel disease: A qualitative interview study. Int J Qual Stud Health Well‐Being. 2019;14(1):1648945. 31382870 10.1080/17482631.2019.1648945 PMC6713182

[40] Limdi JK , Aggarwal D , McLaughlin JT . Dietary practices and beliefs in patients with inflammatory bowel disease. Inflamm Bowel Dis. 2016;22(1):164‐170. 26383912 10.1097/MIB.0000000000000585

