# Supplementary figures and images for: Single‐center experience of the Crohn's disease exclusion diet in the United States: A retrospective study
Source: Nutr Clin Pract. 2026 Apr 15;41(4):1132–43. doi: 10.1002/ncp.70122 (PMC13421003; doi:10.1002/ncp.70122)

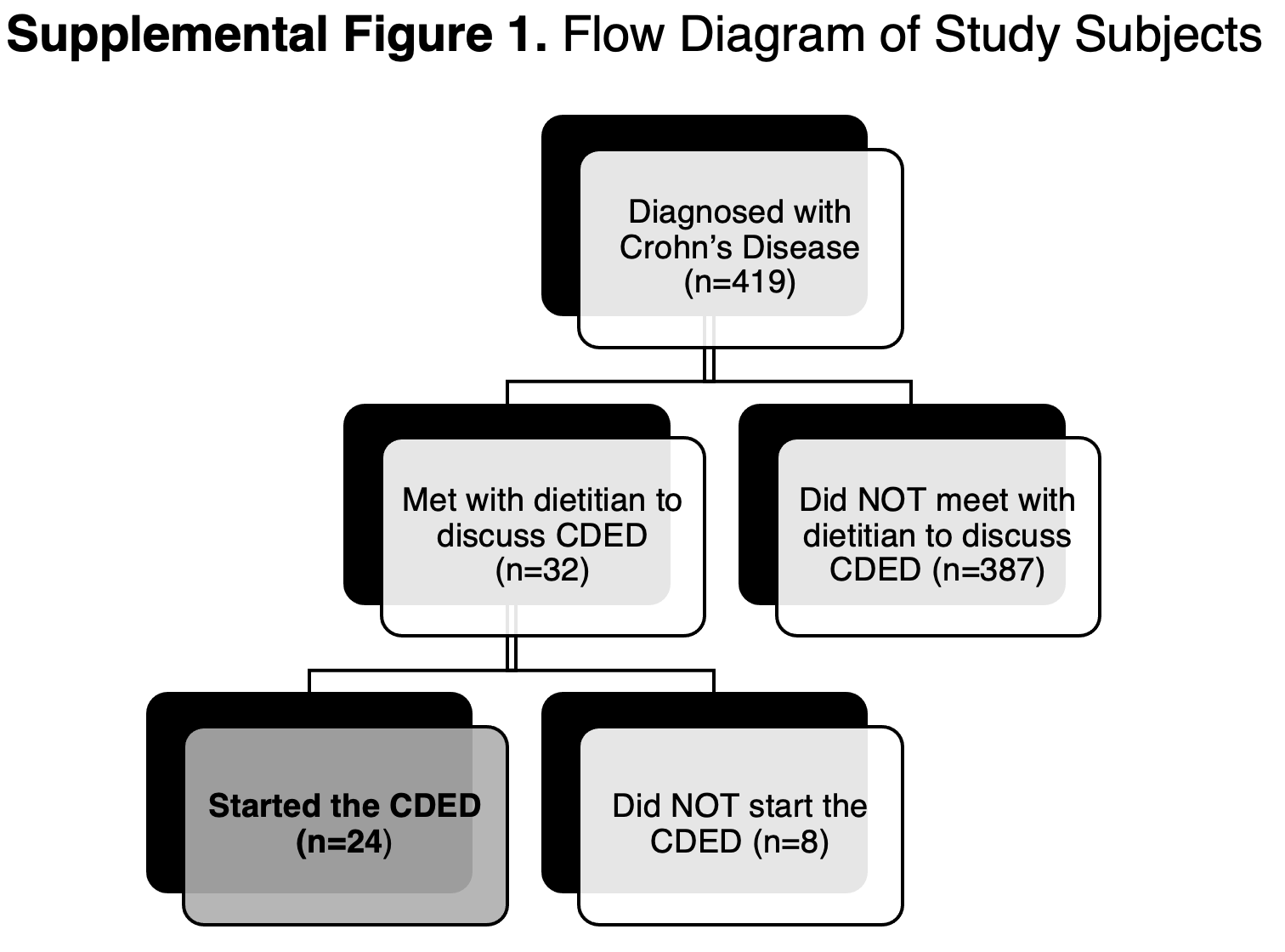

Supplement: Supplementary file 1 — Figure S1: Flow Diagram of Study Subjects. Of note, in those subjects that did not meet with an IBD‐focused dietitian eligibility for the CDED was not assessed. [file NCP-41-1132-s001.tiff]
